# Crystal structure of a copper complex containing the 4-bromo-2-{[(2-hy­droxy­eth­yl)imino]­meth­yl}phenol base Schiff ligand

**DOI:** 10.1107/S2056989026009746

**Published:** 2026-09-30

**Authors:** Adama Niang, Elhadji Sékou Mane, Paolo Pio Mazzeo, Diéwo Ka, Ousmane Diouf, Mohamed Lamine Gaye, Ibrahima Elhadji Thiam, Alessia Bacchi

**Affiliations:** a Département de Chimie Faculté des Sciences et Techniques Université Cheik Anta Diop Dakar Senegal; b Department of Chemical Sciences, Life Science and Environmental Sustainability, University of Parma, Parco Area delle Scienze 17/A, 43124 Parma, Italy; c Biopharmanet-TEC, University of Parma, Parco Area Delle Scienze 27/A, Parma 43124, Italy; Universidade de Sâo Paulo, Brazil

**Keywords:** crystal structure, co-crystal, coordination polymer complex, copper(II)

## Abstract

The coordination co-crystal (4-bromo-2-{[(2-hy­droxy­eth­yl)imino]­meth­yl}pheno­lolato-κ^3^*O*,*N*,*O*′)(thio­cyanato-κ*N*)copper(II)–5-bromo-2-hy­droxy­benz­alde­hyde–water (2/1/1), [Cu(C_9_H_9_BrNO_2_)(NCS)]·0.5C_7_H_5_BrO_2_·0.5H_2_O or [Cu(H*L*)(SCN)]_2_·C_7_H_5_BrO_2_·H_2_O, crystallizes in the monoclinic space group *P*2_1_/*c*. The asymmetric unit contains two mononuclear units of the complex, each consisting of one deprotonated ligand, one thio­cyanate anion (SCN^−^) coordinated through its nitro­gen atom, one free water mol­ecule, and one free 5-bromo­salicyl­aldehyde mol­ecule, forming a co-crystal. The copper atom is four-coordinate, adopting a slightly distorted square-planar geometry defined by the tridentate ligand and the nitro­gen atom of the thio­cyanate anion. Bond length values show that the thio­cyanate ion has a linear S—C≡N arrangement, typical of a terminal, N-bound iso­thio­cyanate group. The water mol­ecule plays a key role in linking the two complex units through O—H⋯O hydrogen bonds. In the crystal, C—H⋯O and C—H⋯Br hydrogen bonds allow the free 5-bromo­salicyl­aldehyde mol­ecule to form a bridge between two adjacent layers. Additional O—H⋯O, C—H⋯S, O—H⋯S, and C—H⋯N inter­actions further consolidate the overall structure.

## Abstract 2

The asymmetric unit contains two mononuclear units of the complex, each consisting of one deprotonated ligand, one thio­cyanate anion (SCN^−^) coordinated through its nitro­gen atom, one free water mol­ecule, and one free 5-bromo­salicyl­aldehyde mol­ecule, forming a co-crystal. The copper atom is four-coordinate, adopting a slightly distorted square-planar geometry defined by the tridentate ligand and the nitro­gen atom of the thio­cyanate anion. The water mol­ecule plays a key role in linking the two complex units through O–H⋯O hydrogen bonds. In the crystal, C—H⋯O and C—H⋯Br hydrogen bonds allow the free 5-bromo­salicyl­aldehyde mol­ecule to form a bridge between two adjacent layers.

## 1. Chemical context

The use of salicyl­aldehyde-derived ligands in the synthesis of coordination complexes has become increasingly important and represents a frequent research topic, particularly in copper-catalysed reactions (Juyal *et al.*, 2023 ▸). Indeed, these ligands contain donor sites such as the phenolic oxygen atom and the imine nitro­gen atom, enabling the formation of stable complexes with various geometries, notably polynuclear copper(II) complexes.

Numerous applications have been reported, including in catalysis (Shelar *et al.*, 2021 ▸), materials science (Abu-Dief & Mohamed, 2015 ▸), and medicine (More *et al.*, 2019 ▸). The formation of a co-crystal associated with a polynuclear copper complex represents an important aspect of supra­molecular chemistry, as such systems are stabilized by networks of non-covalent inter­actions, including hydrogen bonds (Benito *et al.*, 2025 ▸).

In the field of crystal engineering, co-crystals defined as single-phase crystalline materials comprising two or more neutral mol­ecular species have long been exploited to tune physicochemical attributes while preserving the mol­ecular identity of active components (Taylor & Day, 2018 ▸). We have synthesized a tridentate Schiff base ligand, (*E*)-4-bromo-2-{[(2-hy­droxy­eth­yl)imino]­meth­yl}phenol, derived from salicyl­aldehyde, which forms a polynuclear complex with copper(II) chloride and potassium thio­cyanate.
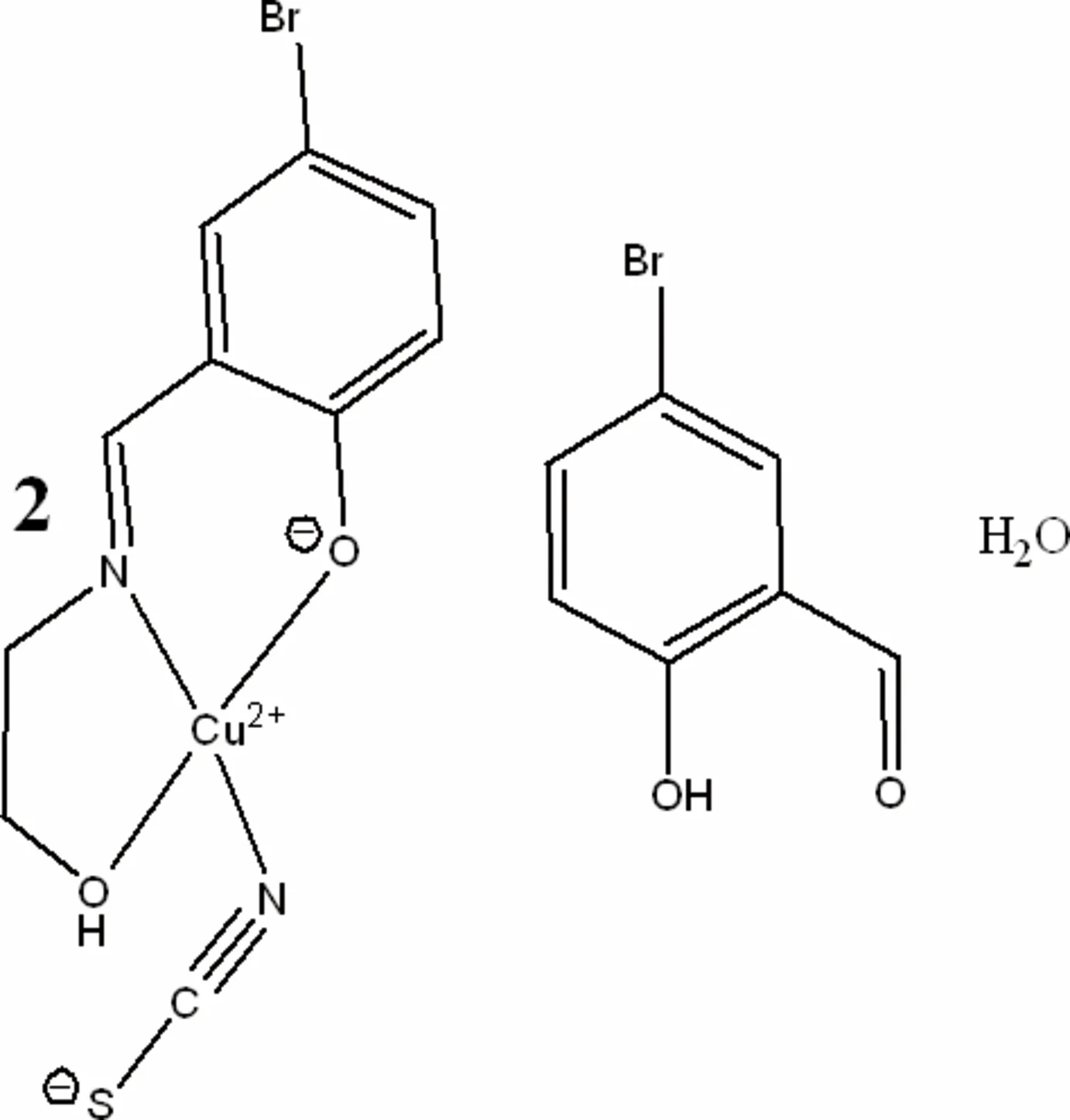


## 2. Structural commentary

**Figure 1:**
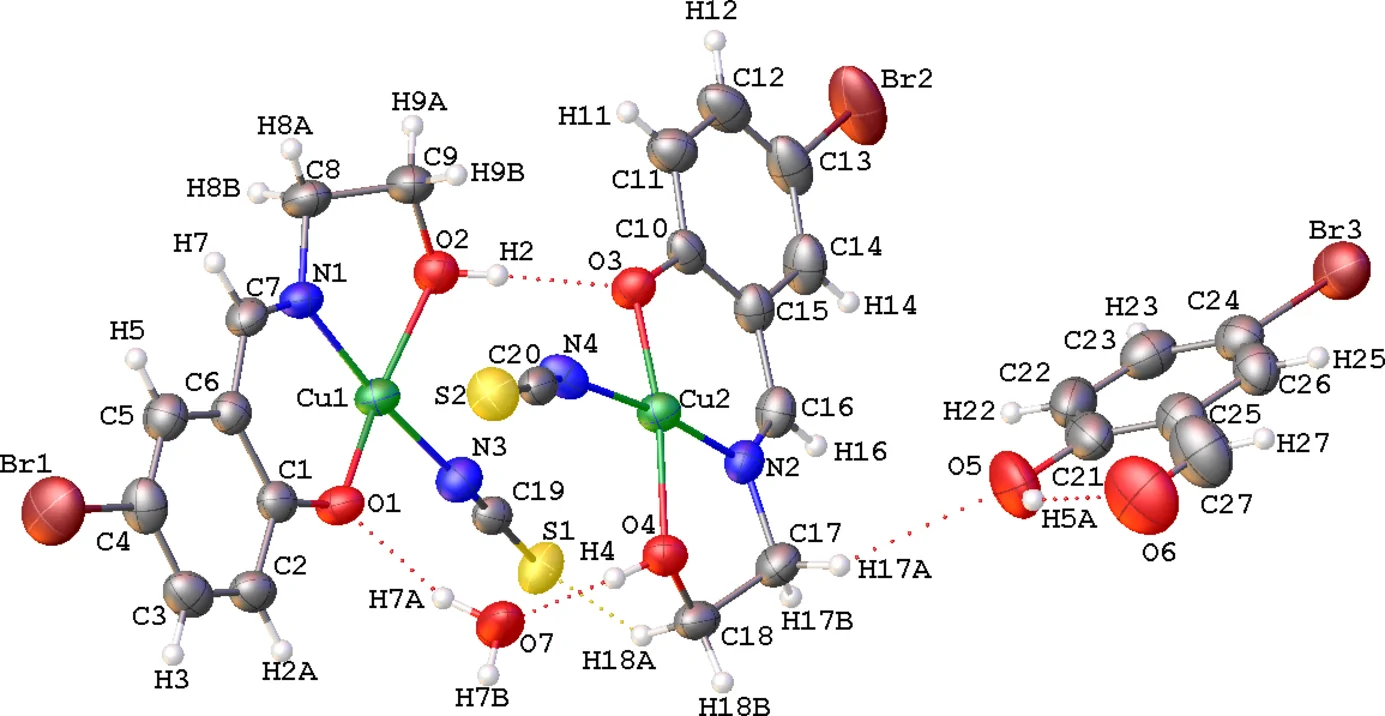
The mol­ecular structure of the title compound showing the atom-numbering scheme. Displacement ellipsoids are drawn at the 50% probability level.

The title compound (Fig. 1 ▸) crystallizes in the monoclinic system, space group *P*2_1_/*c*. The asymmetric unit, [Cu(H*L*)(SCN)]_2_(C_7_H_5_BrO_2_)·H_2_O, contains two monodeprotonated ligand mol­ecules, two independent copper(II) ions, two monodentate thio­cyanate anions, one co-crystallized bromo-hy­droxy­benzaldehyde mol­ecule and one free water mol­ecule.

The Schiff base ligand acts in a tridentate fashion through the phenolate oxygen atom, the azomethine nitro­gen atom, and the alcoholic oxygen atom, resulting in one five-membered (CuOCCN) and one six-membered (CuOCCCN) chelate ring around each Cu^II^ ion.

Each copper atom is four-coordinate, bound by an N_2_O_2_ donor set comprising one azomethine (imine) nitro­gen and two oxygen atoms (one phenolate, one alcoholic) from the Schiff base ligand, together with one nitro­gen atom from its own thio­cyanate group, forming an essentially square-planar, moderately distorted coordination plane. The bond lengths within this square plane are Cu1—O1 = 1.911 (2) Å, Cu1—N1 = 1.928 (3) Å, Cu1—N3 = 1.952 (3) Å, and Cu1—O2 = 2.038 (2) Å. For the corresponding values for Cu2, see Table 1 ▸).

A single, additional axial contact to the nitro­gen atom of the thio­cyanate group of the neighbouring mononuclear unit is present at each copper centre, but this contact is long and markedly asymmetric between the two independent centres [2.961 (3) Å for Cu1 *versus* 3.100 (3) Å for Cu2]. The latter value exceeds the sum of the van der Waals radii for Cu⋯N and cannot reasonably be described as a coordination bond. The geometry is therefore described as distorted square-planar (four-coordinate, N_2_O_2_), with a weak, asymmetric secondary axial contact of semi-coordinate character, rather than as distorted square-planar.

It is this weak, asymmetric N⋯Cu axial contact, not a Cu—S bond, that links each mononuclear [Cu(H*L*)(SCN)] unit to its neighbour and propagates the one-dimensional polymeric chain.

The deviations of the angles from the ideal value of 90° [O1—Cu1—N1 = 93.58 (10)°, O2—Cu1—N3 = 89.45 (11)°, N1—Cu1—O2 = 82.51 (10)°, and O1—Cu1—N3 = 94.69 (11)°] confirm the distortion of the coordination geometry.

The Cu1> ion is displaced from the least-squares plane defined by O1, N1, O2 and N3 (r.m.s. deviation 0.117 Å) by 0.006 (12) Å, indicating that the metal centre lies essentially within the equatorial plane. The dihedral angle of 175.63 (17)° between the phenolate ring plane (O1/C1–C6) and the plane of the open-chain fragment (C7/N1/C8/C9/O2) reveals a slight but measurable deviation from planarity, indicative of a twist in the ligand backbone.

The Cu1⋯Cu2 separation between two adjacent units is 3.7765 (5) Å, a distance too long to invoke any meaningful metal–metal inter­action. The *cis* angles span the range 82.51 (10)–94.69 (11)°, in good agreement with values reported for related complexes. The *trans* angles O1—Cu1—O2 = 166.82 (10)° and N3—Cu1—N1 = 171.73 (12)° deviate significantly from the ideal value of 180°, further confirming that Cu1 resides in a severely distorted square-planar coordination environment. The environment about Cu2 is similar, see Table 1 ▸).

The bond lengths within the two thio­cyanate groups are S1—C19 = 1.629 (3) Å, C19—N3 = 1.153 (4) Å, and S2—C20 = 1.643 (4) Å, C20—N4 = 1.106 (4) Å. These values are consistent with the linear S—C≡N arrangement typical of a terminal, N-bound iso­thio­cyanate group.

The sulfur atoms S1 and S2 are not bonded to any copper atom. The bridging mode is therefore not the classical μ-1,3 (S,N) mode frequently found in Cu–SCN polymers, but an asymmetric end-on μ-1,1 (N,N′) bridge, in which each thio­cyanate nitro­gen atom is strongly bonded to its own copper centre and forms a second, much weaker, semi-coordinate contact with the neighbouring copper centre, as summarized below.

## 3. Supra­molecular features

**Figure 2:**
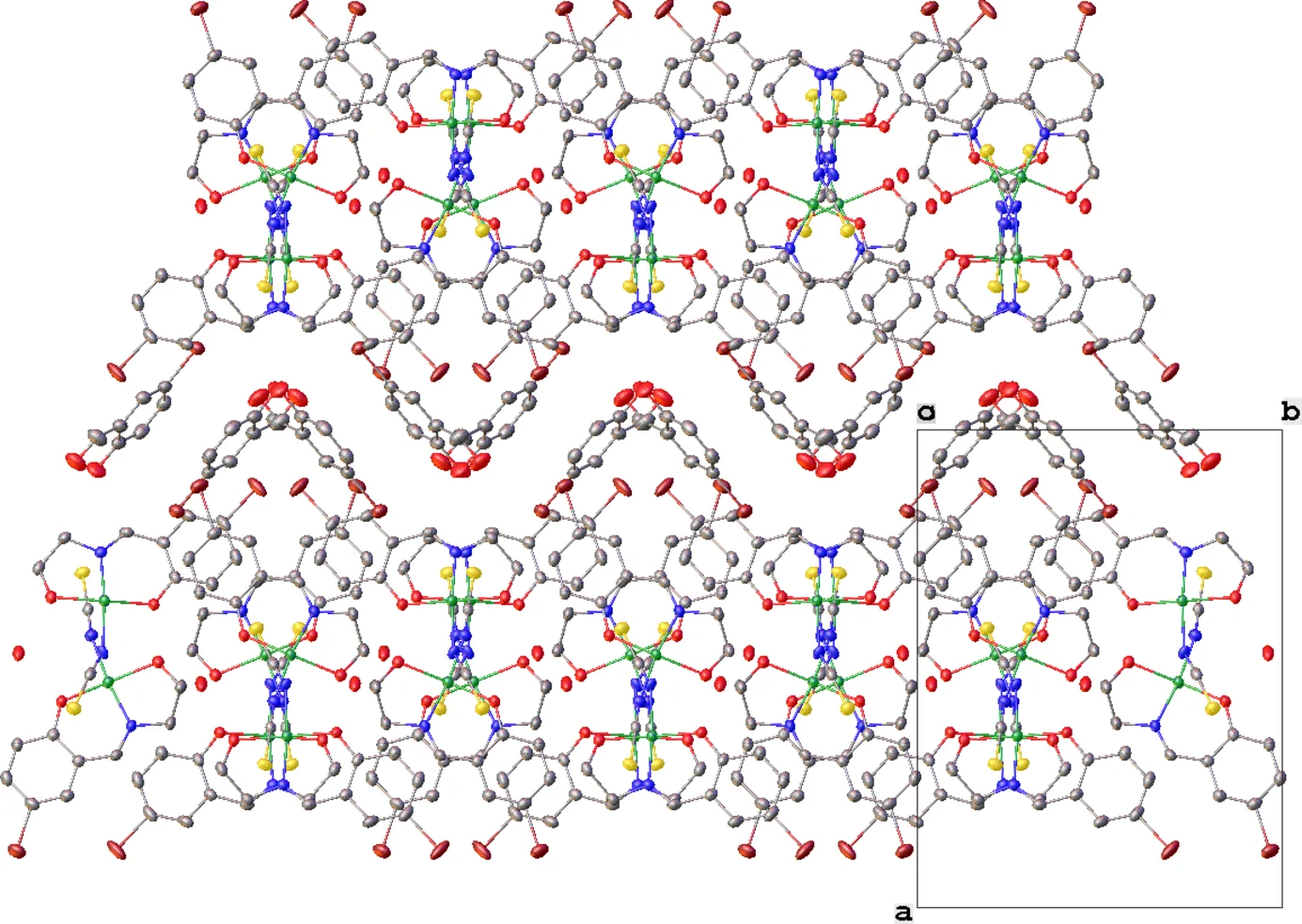
Partial packing diagram for the title compound along [001].

We note four strong inter­actions of the O—H—O type (Table 2 ▸). The water mol­ecule connects unit Cu2 *via* O4—H4⋯O7 to unit Cu1 *via* O7—H7*A*··O1. The O2—H2⋯O3 hydrogen bond similarly connects the two units. The O5—H5*A*⋯O6 inter­action corresponds to the classical intra­molecular hydrogen bond within the co-crystallized aldehyde fragment, confirming its nature as an isolated unit maintained in the network mainly by the weak C—H⋯·Br, C—H⋯S, C—H⋯N and C—H⋯O contacts and extending the three-dimensional network and further enhances the cohesion of the structure. The packing is shown in Fig. 2 ▸.

## 4. Database survey

A search of the Cambridge Structural Database (CSD version 5.46, updates of November 2024 and February 2025; Groom *et al.*, 2016) yielded 25 hits containing crystal structures of coordination complexes bearing the 4-bromo-2-{[(2-hy­droxy­eth­yl)imino]­meth­yl}phenol ligand. The first result corresponds to the structure of the free ligand reported by Thakur *et al.* 2024 ▸ (refcode YAWNOB01). 23 of the remaining hits correspond to coordination complexes of first-row transition metals. Only one second-row transition metal complex is noted, namely a cadmium complex (AWOHIE; Yu *et al.*, 2011 ▸). Half of the hits correspond to mononuclear complexes in which two mol­ecules of the tridentate ligand chelate the metal ion including refcodes BAVGUD, BAVROI (Abbasi *et al.*, 2017 ▸); HEDKUX (Liang *et al.*, 2006 ▸); HEDKUX01 (Chumakov *et al.*, 2007 ▸); HEDKUX02 (Zabierowski *et al.*, 2013 ▸); MIWSEQ (De *et al.*, 2001 ▸), among others. Polynuclear complexes are also reported, including a dinuclear copper complex (FOQKAY; Thakurta *et al.*, 2009*a* ▸,*b* ▸), a tetra­nuclear copper complex (LICWAW; Yoshida *et al.*), and a tetra­nuclear iron complex (EBUWII; Oshio *et al.*, 2004 ▸).

## 5. Synthesis and crystallization

The Schiff base ligand H*L* was prepared by adding 2.5 g (13.5 mmol) of 5-bromo­salicyl­aldehyde and 0.8 g (13.5 mmol) of 2-amino­ethanol, along with a few drops of glacial acetic acid, to 20 mL of ethanol in a flask. The mixture was refluxed, then evaporated to dryness using a rotary evaporator, giving a light-brown oil in 80% yield. This oil was dissolved in ethanol (100 mL) to give a 0.108 mol l^−1^ ligand solution.

For the complex synthesis, 0.17 g (1 mmol) of copper(II) chloride dihydrate and 0.19 g (2 mmol) of potassium thio­cyanate were dissolved in 10 mL of methanol and stirred under reflux for 30 minutes, giving a brown solution. Then, 9.2 mL of the ligand solution was added dropwise to the metal solution. After refluxing for 1 h, a green precipitate formed, which was filtered, washed with methanol, and dried, giving 0.14 g of product. Green single crystals suitable for X-ray diffraction were obtained after two weeks.

## 6. Refinement

Crystal data, data collection and structure refinement details are summarized in Table 3 ▸. H atoms were positioned geometrically and refined as riding with *U*_iso_(H) = 1.2*U*_eq_(C).

## Supplementary Material

Crystal structure: contains datablock(s) I. DOI: 10.1107/S2056989026009746/ex2102sup1.cif

Structure factors: contains datablock(s) I. DOI: 10.1107/S2056989026009746/ex2102Isup2.hkl

CCDC reference: 2588876

Additional supporting information:  crystallographic information; 3D view; checkCIF report

## Figures and Tables

**Table 1:** Selected geometric parameters (Å, °)

Cu1—O1	1.911 (2)	Cu2—O3	1.923 (2)
Cu1—O2	2.038 (2)	Cu2—O4	2.005 (2)
Cu1—N1	1.928 (3)	Cu2—N4	1.995 (3)
Cu1—N3	1.952 (3)	Cu2—N2	1.927 (3)
			
O1—Cu1—O2	166.82 (10)	O3—Cu2—O4	166.23 (10)
N3—Cu1—O2	89.45 (11)	O3—Cu2—N4	91.49 (10)
O1—Cu1—N1	93.58 (10)	O3—Cu2—N2	93.35 (11)
O1—Cu1—N3	94.69 (11)	N4—Cu2—O4	92.01 (10)
N1—Cu1—O2	82.51 (10)	N2—Cu2—O4	82.24 (11)
N1—Cu1—N3	171.73 (12)	N2—Cu2—N4	173.38 (11)

**Table 2:** Hydrogen-bond geometry (Å, °)

*D*—H⋯*A*	*D*—H	H⋯*A*	*D*⋯*A*	*D*—H⋯*A*
O4—H4⋯O7	0.82	1.75	2.562 (4)	174
O2—H2⋯O3	0.82	1.84	2.647 (3)	169
O7—H7*A*⋯O1	0.85	1.88	2.709 (4)	163
O7—H7*B*⋯S2^i^	0.85	2.64	3.290 (3)	135
O5—H5*A*⋯O6	0.82	1.94	2.655 (6)	146
C18—H18*A*⋯Br3^ii^	0.97	3.32	3.856 (4)	117
C18—H18*A*⋯S1	0.97	2.98	3.801 (4)	143
C18—H18*A*⋯N3	0.97	3.22	3.785 (5)	119
C17—H17*A*⋯O5	0.97	2.73	3.368 (5)	124
C16—H16⋯O6^iii^	0.93	2.52	3.376 (5)	153
C2—H2*A*⋯O2^iv^	0.93	2.92	3.726 (4)	146
C2—H2*A*⋯O7	0.93	3.08	3.548 (5)	113
C9—H9*A*⋯N4^v^	0.97	3.20	3.851 (4)	126
C9—H9*B*⋯O3^v^	0.97	3.03	3.613 (5)	120
C9—H9*B*⋯N4	0.97	3.20	3.700 (5)	114
C11—H11⋯S2^v^	0.93	3.11	3.709 (4)	124
C8—H8*A*⋯S1^vi^	0.97	3.29	3.881 (3)	121
C8—H8*A*⋯O3^v^	0.97	3.07	3.773 (5)	130
C8—H8*B*⋯S2^iii^	0.97	3.30	3.784 (4)	113
C8—H8*B*⋯O7^vi^	0.97	2.96	3.739 (6)	138
C14—H14⋯O6^iii^	0.93	3.12	3.827 (6)	134
C12—H12⋯S2^v^	0.93	2.93	3.616 (5)	131
C22—H22⋯O6^iii^	0.93	2.97	3.847 (6)	158
C27—H27⋯Br2^vii^	0.93	3.04	3.949 (6)	166

Symmetry codes: (i) 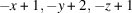; (ii) 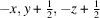; (iii) 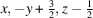; (iv) 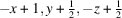; (v) 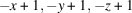; (vi) 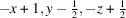; (vii) 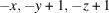.

**Table 3:** Experimental details

Crystal data	
Chemical formula	[Cu(C_9_H_9_BrNO_2_)(NCS)]·0.5C_7_H_5_BrO_2_·0.5H_2_O
*M*_r_	948.44
Crystal system, space group	Monoclinic, *P*2_1_/*c*
Temperature (K)	296
*a*, *b*, *c* (Å)	18.4400 (5), 13.7346 (3), 13.6402 (3)
β (°)	103.136 (1)
*V* (Å^3^)	3364.20 (14)
*Z*	4
Radiation type	Mo *K*α
μ (mm^−1^)	5.00
Crystal size (mm)	0.2 × 0.2 × 0.2

Data collection	
Diffractometer	Bruker D8 Venture Equipped with PhotonII Detector
Absorption correction	Multi-scan (*SADABS*; Krause *et al.*, 2015 ▸)
No. of measured, independent and observed [*I* > 2σ(*I*)] reflections	60122, 6909, 5316
*R*_int_	0.070
(sin θ/λ)_max_ (Å^−1^)	0.626

Refinement	
*R*[*F*^2^ > 2σ(*F*^2^)], *wR*(*F*^2^), *S*	0.034, 0.081, 1.01
No. of reflections	6909
No. of parameters	412
H-atom treatment	H-atom parameters constrained
Δρ_max_, Δρ_min_ (e Å^−3^)	0.62, −0.61

Computer programs: *APEX* and *SAINT* (Bruker, 2014 ▸), *OLEX2.solve* (Bourhis *et al.*, 2015), *SHELXL2018/3* (Sheldrick, 2015 ▸) and *OLEX2* (Dolomanov *et al.*, 2009 ▸).

## supplementary crystallographic information

### (4-Bromo-2-{[(2-hydroxyethyl)imino]methyl}phenololato-κ3O,N,O')(thiocyanato-κN)copper(II)–5-bromo-2-hydroxybenzaldehyde–water (2/1/1) Crystal data

**Table:**

[Cu(C_9_H_9_BrNO_2_)(NCS)]·0.5C_7_H_5_BrO_2_·0.5H_2_O	*F*(000) = 1864
*M**_r_* = 948.44	*D*_x_ = 1.873 Mg m^−^^3^
Monoclinic, *P*2_1_/*c*	Mo *K*α radiation, λ = 0.71073 Å
*a* = 18.4400 (5) Å	Cell parameters from 60122 reflections
*b* = 13.7346 (3) Å	θ = 1.9–26.4°
*c* = 13.6402 (3) Å	µ = 5.00 mm^−^^1^
β = 103.136 (1)°	*T* = 296 K
*V* = 3364.20 (14) Å^3^	Prism, green
*Z* = 4	0.2 × 0.2 × 0.2 mm

### (4-Bromo-2-{[(2-hydroxyethyl)imino]methyl}phenololato-κ3O,N,O')(thiocyanato-κN)copper(II)–5-bromo-2-hydroxybenzaldehyde–water (2/1/1) Data collection

**Table:**

Bruker D8 Venture Equipped with PhotonII Detector diffractometer	5316 reflections with *I* > 2σ(*I*)
profile data from ω–scans	*R*_int_ = 0.070
Absorption correction: multi-scan (SADABS; Krause *et al.*, 2015)	θ_max_ = 26.4°, θ_min_ = 1.9°
	*h* = −23→23
60122 measured reflections	*k* = −17→17
6909 independent reflections	*l* = −17→15

### (4-Bromo-2-{[(2-hydroxyethyl)imino]methyl}phenololato-κ3O,N,O')(thiocyanato-κN)copper(II)–5-bromo-2-hydroxybenzaldehyde–water (2/1/1) Refinement

**Table:**

Refinement on *F*^2^	0 restraints
Least-squares matrix: full	Hydrogen site location: mixed
*R*[*F*^2^ > 2σ(*F*^2^)] = 0.034	H-atom parameters constrained
*wR*(*F*^2^) = 0.081	*w* = 1/[σ^2^(*F*_o_^2^) + (0.0255*P*)^2^ + 4.7568*P*] where *P* = (*F*_o_^2^ + 2*F*_c_^2^)/3
*S* = 1.01	(Δ/σ)_max_ = 0.001
6909 reflections	Δρ_max_ = 0.62 e Å^−^^3^
412 parameters	Δρ_min_ = −0.61 e Å^−^^3^

### (4-Bromo-2-{[(2-hydroxyethyl)imino]methyl}phenololato-κ3O,N,O')(thiocyanato-κN)copper(II)–5-bromo-2-hydroxybenzaldehyde–water (2/1/1) Special details

**Table:**

Geometry. All esds (except the esd in the dihedral angle between two l.s. planes) are estimated using the full covariance matrix. The cell esds are taken into account individually in the estimation of esds in distances, angles and torsion angles; correlations between esds in cell parameters are only used when they are defined by crystal symmetry. An approximate (isotropic) treatment of cell esds is used for estimating esds involving l.s. planes.

### (4-Bromo-2-{[(2-hydroxyethyl)imino]methyl}phenololato-κ3O,N,O')(thiocyanato-κN)copper(II)–5-bromo-2-hydroxybenzaldehyde–water (2/1/1) Fractional atomic coordinates and isotropic or equivalent isotropic displacement parameters (Å^2^)

**Table:**

	*x*	*y*	*z*	*U*_iso_*/*U*_eq_	
Cu2	0.35837 (2)	0.72579 (3)	0.41858 (3)	0.03257 (10)	
Cu1	0.52874 (2)	0.71541 (3)	0.30893 (3)	0.03342 (10)	
Br3	−0.17062 (2)	0.47243 (3)	0.42161 (4)	0.06458 (14)	
Br1	0.88193 (3)	0.96575 (4)	0.53387 (5)	0.07697 (16)	
Br2	0.11977 (3)	0.30742 (4)	0.22273 (4)	0.08438 (19)	
S1	0.29927 (5)	0.78586 (7)	0.09224 (7)	0.0424 (2)	
S2	0.58322 (6)	0.80940 (7)	0.62694 (8)	0.0485 (2)	
O3	0.36588 (12)	0.59043 (15)	0.38633 (18)	0.0365 (5)	
O4	0.35052 (13)	0.87140 (16)	0.41724 (18)	0.0386 (5)	
H4	0.388208	0.901725	0.414008	0.058*	
O1	0.56911 (13)	0.84305 (15)	0.33838 (18)	0.0376 (5)	
O2	0.48631 (13)	0.57876 (16)	0.31199 (19)	0.0399 (6)	
H2	0.446357	0.578002	0.328833	0.060*	
N4	0.46559 (16)	0.73030 (19)	0.4895 (2)	0.0353 (6)	
N1	0.61991 (15)	0.65156 (18)	0.3749 (2)	0.0340 (6)	
N2	0.25666 (15)	0.7357 (2)	0.3433 (2)	0.0338 (6)	
N3	0.43073 (16)	0.7612 (2)	0.2376 (2)	0.0397 (7)	
O7	0.46760 (17)	0.96055 (19)	0.3943 (3)	0.0702 (10)	
H7A	0.502551	0.934333	0.372361	0.105*	
H7B	0.481742	1.019032	0.407084	0.105*	
O5	0.0839 (2)	0.7473 (3)	0.4295 (3)	0.0837 (11)	
H5A	0.093367	0.779032	0.481825	0.126*	
C19	0.37653 (18)	0.7704 (2)	0.1765 (2)	0.0305 (7)	
C1	0.63814 (19)	0.8652 (2)	0.3819 (2)	0.0336 (7)	
C6	0.69389 (18)	0.7959 (2)	0.4217 (2)	0.0339 (7)	
C7	0.68112 (18)	0.6930 (2)	0.4166 (3)	0.0354 (7)	
H7	0.720737	0.653006	0.446251	0.042*	
C10	0.30904 (19)	0.5317 (2)	0.3505 (2)	0.0345 (7)	
C18	0.2894 (2)	0.9050 (2)	0.3404 (3)	0.0412 (8)	
H18A	0.302755	0.905842	0.275613	0.049*	
H18B	0.275062	0.970303	0.355478	0.049*	
C17	0.2264 (2)	0.8346 (2)	0.3389 (3)	0.0424 (8)	
H17A	0.204564	0.846199	0.396192	0.051*	
H17B	0.188028	0.843103	0.277853	0.051*	
O6	0.0669 (2)	0.8034 (3)	0.6092 (3)	0.0965 (12)	
C15	0.23619 (19)	0.5640 (3)	0.3092 (3)	0.0376 (8)	
C16	0.21500 (19)	0.6653 (3)	0.3038 (3)	0.0385 (8)	
H16	0.166982	0.680496	0.268639	0.046*	
C2	0.6592 (2)	0.9643 (2)	0.3897 (3)	0.0399 (8)	
H2A	0.623644	1.011294	0.363949	0.048*	
C20	0.5114 (2)	0.7642 (2)	0.5457 (3)	0.0385 (8)	
C5	0.7662 (2)	0.8277 (3)	0.4681 (3)	0.0426 (8)	
H5	0.802604	0.782312	0.495451	0.051*	
C9	0.5384 (2)	0.5164 (3)	0.3777 (3)	0.0470 (9)	
H9A	0.528637	0.448840	0.358511	0.056*	
H9B	0.533871	0.524355	0.446689	0.056*	
C11	0.3229 (2)	0.4310 (3)	0.3530 (3)	0.0450 (9)	
H11	0.370795	0.408127	0.379841	0.054*	
C8	0.6145 (2)	0.5446 (2)	0.3682 (3)	0.0452 (9)	
H8A	0.651666	0.515002	0.421687	0.054*	
H8B	0.622919	0.522553	0.304106	0.054*	
C3	0.7297 (2)	0.9935 (3)	0.4337 (3)	0.0446 (9)	
H3	0.741978	1.059317	0.437188	0.054*	
C4	0.7831 (2)	0.9240 (3)	0.4734 (3)	0.0461 (9)	
C14	0.1799 (2)	0.4965 (3)	0.2705 (3)	0.0495 (10)	
H14	0.131914	0.517809	0.241540	0.059*	
C24	−0.0898 (2)	0.5597 (3)	0.4264 (3)	0.0462 (9)	
C13	0.1959 (2)	0.3992 (3)	0.2754 (3)	0.0521 (10)	
C12	0.2665 (2)	0.3655 (3)	0.3162 (3)	0.0518 (10)	
H12	0.276256	0.299005	0.319076	0.062*	
C25	−0.0669 (2)	0.6183 (3)	0.5070 (3)	0.0488 (9)	
H25	−0.090676	0.615755	0.560213	0.059*	
C26	−0.0078 (2)	0.6828 (3)	0.5107 (3)	0.0511 (10)	
C21	0.0275 (2)	0.6859 (3)	0.4308 (4)	0.0555 (11)	
C23	−0.0552 (2)	0.5622 (3)	0.3469 (3)	0.0559 (11)	
H23	−0.071640	0.521800	0.291633	0.067*	
C22	0.0035 (2)	0.6245 (3)	0.3492 (4)	0.0611 (12)	
H22	0.027240	0.625415	0.295851	0.073*	
C27	0.0160 (3)	0.7449 (4)	0.5975 (4)	0.0801 (15)	
H27	−0.009566	0.739894	0.648683	0.096*	

### (4-Bromo-2-{[(2-hydroxyethyl)imino]methyl}phenololato-κ3O,N,O')(thiocyanato-κN)copper(II)–5-bromo-2-hydroxybenzaldehyde–water (2/1/1) Atomic displacement parameters (Å^2^)

**Table:**

	*U*^11^	*U*^22^	*U*^33^	*U*^12^	*U*^13^	*U*^23^
Cu2	0.0329 (2)	0.0294 (2)	0.0334 (2)	−0.00098 (16)	0.00347 (17)	−0.00299 (17)
Cu1	0.0343 (2)	0.0288 (2)	0.0348 (2)	0.00313 (16)	0.00299 (17)	0.00019 (17)
Br3	0.0459 (2)	0.0590 (3)	0.0845 (3)	−0.0085 (2)	0.0057 (2)	−0.0066 (2)
Br1	0.0490 (3)	0.0770 (3)	0.0974 (4)	−0.0180 (2)	0.0010 (2)	−0.0192 (3)
Br2	0.0856 (4)	0.0834 (3)	0.0933 (4)	−0.0563 (3)	0.0395 (3)	−0.0404 (3)
S1	0.0369 (5)	0.0564 (6)	0.0320 (5)	0.0095 (4)	0.0035 (4)	−0.0040 (4)
S2	0.0446 (5)	0.0535 (6)	0.0441 (5)	−0.0064 (4)	0.0029 (4)	−0.0037 (4)
O3	0.0330 (12)	0.0300 (11)	0.0454 (14)	−0.0018 (9)	0.0066 (10)	−0.0056 (10)
O4	0.0419 (14)	0.0305 (12)	0.0446 (14)	−0.0008 (10)	0.0124 (11)	−0.0016 (11)
O1	0.0366 (13)	0.0297 (11)	0.0434 (14)	0.0029 (10)	0.0026 (11)	−0.0007 (10)
O2	0.0391 (14)	0.0338 (12)	0.0475 (15)	−0.0012 (11)	0.0111 (11)	−0.0019 (11)
N4	0.0459 (17)	0.0333 (15)	0.0233 (14)	−0.0138 (13)	0.0007 (13)	−0.0050 (12)
N1	0.0369 (16)	0.0277 (13)	0.0372 (16)	0.0042 (11)	0.0075 (12)	0.0027 (12)
N2	0.0313 (15)	0.0385 (15)	0.0319 (15)	0.0023 (12)	0.0078 (12)	−0.0005 (12)
N3	0.0369 (16)	0.0424 (16)	0.0379 (17)	0.0038 (13)	0.0044 (14)	0.0008 (13)
O7	0.0595 (19)	0.0332 (14)	0.131 (3)	0.0022 (13)	0.048 (2)	0.0022 (17)
O5	0.063 (2)	0.084 (3)	0.112 (3)	−0.0221 (18)	0.038 (2)	0.012 (2)
C19	0.0388 (19)	0.0279 (15)	0.0267 (16)	0.0020 (13)	0.0114 (15)	−0.0019 (13)
C1	0.0390 (19)	0.0350 (17)	0.0289 (17)	0.0013 (14)	0.0123 (15)	0.0002 (14)
C6	0.0345 (18)	0.0370 (17)	0.0307 (17)	0.0011 (14)	0.0086 (14)	−0.0002 (14)
C7	0.0327 (18)	0.0366 (17)	0.0365 (19)	0.0083 (14)	0.0069 (15)	0.0067 (15)
C10	0.0414 (19)	0.0331 (17)	0.0327 (18)	−0.0085 (14)	0.0161 (15)	−0.0065 (14)
C18	0.051 (2)	0.0343 (18)	0.041 (2)	0.0109 (16)	0.0169 (17)	0.0069 (16)
C17	0.037 (2)	0.0419 (19)	0.048 (2)	0.0089 (15)	0.0089 (16)	0.0030 (17)
O6	0.078 (3)	0.102 (3)	0.099 (3)	−0.041 (2)	−0.001 (2)	−0.012 (2)
C15	0.0362 (19)	0.0426 (19)	0.0358 (19)	−0.0062 (15)	0.0117 (15)	−0.0088 (15)
C16	0.0281 (17)	0.050 (2)	0.0364 (19)	−0.0015 (15)	0.0051 (14)	−0.0060 (16)
C2	0.049 (2)	0.0314 (17)	0.041 (2)	0.0011 (15)	0.0136 (17)	−0.0020 (15)
C20	0.044 (2)	0.0340 (17)	0.043 (2)	0.0084 (16)	0.0222 (18)	0.0087 (16)
C5	0.0369 (19)	0.047 (2)	0.044 (2)	0.0020 (16)	0.0080 (16)	−0.0002 (17)
C9	0.048 (2)	0.0324 (18)	0.063 (3)	0.0054 (16)	0.0182 (19)	0.0097 (18)
C11	0.052 (2)	0.0371 (19)	0.046 (2)	−0.0023 (16)	0.0118 (18)	−0.0015 (16)
C8	0.046 (2)	0.0290 (17)	0.061 (3)	0.0065 (15)	0.0132 (18)	0.0037 (17)
C3	0.057 (2)	0.0362 (19)	0.042 (2)	−0.0074 (17)	0.0153 (18)	−0.0066 (16)
C4	0.040 (2)	0.053 (2)	0.044 (2)	−0.0118 (17)	0.0093 (17)	−0.0111 (18)
C14	0.037 (2)	0.060 (2)	0.053 (2)	−0.0152 (18)	0.0127 (18)	−0.018 (2)
C24	0.037 (2)	0.046 (2)	0.053 (2)	0.0041 (16)	0.0040 (18)	0.0042 (18)
C13	0.060 (3)	0.052 (2)	0.051 (2)	−0.027 (2)	0.025 (2)	−0.0200 (19)
C12	0.073 (3)	0.0360 (19)	0.052 (2)	−0.0137 (19)	0.024 (2)	−0.0082 (18)
C25	0.040 (2)	0.058 (2)	0.047 (2)	−0.0010 (18)	0.0089 (17)	0.0069 (19)
C26	0.041 (2)	0.057 (2)	0.053 (2)	−0.0026 (18)	0.0039 (18)	0.005 (2)
C21	0.041 (2)	0.056 (2)	0.070 (3)	0.0054 (19)	0.013 (2)	0.017 (2)
C23	0.060 (3)	0.052 (2)	0.054 (3)	0.012 (2)	0.009 (2)	0.001 (2)
C22	0.060 (3)	0.065 (3)	0.066 (3)	0.019 (2)	0.031 (2)	0.019 (2)
C27	0.069 (3)	0.093 (4)	0.074 (4)	−0.030 (3)	0.008 (3)	−0.006 (3)

### (4-Bromo-2-{[(2-hydroxyethyl)imino]methyl}phenololato-κ3O,N,O')(thiocyanato-κN)copper(II)–5-bromo-2-hydroxybenzaldehyde–water (2/1/1) Geometric parameters (Å, º)

**Table:**

Cu1—O1	1.911 (2)	C18—H18B	0.9700
Cu1—O2	2.038 (2)	C18—C17	1.508 (5)
Cu1—N1	1.928 (3)	C17—H17A	0.9700
Cu1—N3	1.952 (3)	C17—H17B	0.9700
Cu2—O3	1.923 (2)	O6—C27	1.217 (5)
Cu2—O4	2.005 (2)	C15—C16	1.442 (5)
Cu2—N4	1.995 (3)	C15—C14	1.403 (5)
Cu2—N2	1.927 (3)	C16—H16	0.9300
Br3—C24	1.903 (4)	C2—H2A	0.9300
Br1—C4	1.910 (4)	C2—C3	1.363 (5)
Br2—C13	1.902 (4)	C5—H5	0.9300
S1—C19	1.629 (3)	C5—C4	1.358 (5)
S2—C20	1.643 (4)	C9—H9A	0.9700
O3—C10	1.325 (4)	C9—H9B	0.9700
O4—H4	0.8200	C9—C8	1.490 (5)
O4—C18	1.430 (4)	C11—H11	0.9300
O1—C1	1.312 (4)	C11—C12	1.380 (5)
O2—H2	0.8200	C8—H8A	0.9700
O2—C9	1.437 (4)	C8—H8B	0.9700
N4—C20	1.106 (4)	C3—H3	0.9300
N1—C7	1.277 (4)	C3—C4	1.390 (5)
N1—C8	1.474 (4)	C14—H14	0.9300
N2—C17	1.464 (4)	C14—C13	1.367 (6)
N2—C16	1.276 (4)	C24—C25	1.350 (5)
N3—C19	1.153 (4)	C24—C23	1.378 (6)
O7—H7A	0.8502	C13—C12	1.375 (6)
O7—H7B	0.8501	C12—H12	0.9300
O5—H5A	0.8200	C25—H25	0.9300
O5—C21	1.343 (5)	C25—C26	1.396 (5)
C1—C6	1.416 (5)	C26—C21	1.391 (6)
C1—C2	1.412 (5)	C26—C27	1.445 (7)
C6—C7	1.431 (5)	C21—C22	1.387 (6)
C6—C5	1.409 (5)	C23—H23	0.9300
C7—H7	0.9300	C23—C22	1.375 (6)
C10—C15	1.405 (5)	C22—H22	0.9300
C10—C11	1.406 (5)	C27—H27	0.9300
C18—H18A	0.9700		
			
O1—Cu1—O2	166.82 (10)	C15—C16—H16	117.3
N3—Cu1—O2	89.45 (11)	C1—C2—H2A	118.8
O1—Cu1—N1	93.58 (10)	C3—C2—C1	122.4 (3)
O1—Cu1—N3	94.69 (11)	C3—C2—H2A	118.8
N1—Cu1—O2	82.51 (10)	N4—C20—S2	176.2 (3)
N1—Cu1—N3	171.73 (12)	C6—C5—H5	119.7
O3—Cu2—O4	166.23 (10)	C4—C5—C6	120.6 (3)
O3—Cu2—N4	91.49 (10)	C4—C5—H5	119.7
O3—Cu2—N2	93.35 (11)	O2—C9—H9A	110.2
N4—Cu2—O4	92.01 (10)	O2—C9—H9B	110.2
N2—Cu2—O4	82.24 (11)	O2—C9—C8	107.5 (3)
N2—Cu2—N4	173.38 (11)	H9A—C9—H9B	108.5
C10—O3—Cu2	125.5 (2)	C8—C9—H9A	110.2
Cu2—O4—H4	116.5	C8—C9—H9B	110.2
C18—O4—Cu2	111.8 (2)	C10—C11—H11	119.5
C18—O4—H4	109.5	C12—C11—C10	121.0 (4)
C1—O1—Cu1	126.8 (2)	C12—C11—H11	119.5
Cu1—O2—H2	113.0	N1—C8—C9	107.8 (3)
C9—O2—Cu1	110.9 (2)	N1—C8—H8A	110.1
C9—O2—H2	109.5	N1—C8—H8B	110.1
C20—N4—Cu2	150.0 (3)	C9—C8—H8A	110.1
C7—N1—Cu1	126.4 (2)	C9—C8—H8B	110.1
C7—N1—C8	120.9 (3)	H8A—C8—H8B	108.5
C8—N1—Cu1	112.6 (2)	C2—C3—H3	120.3
C17—N2—Cu2	113.8 (2)	C2—C3—C4	119.3 (3)
C16—N2—Cu2	126.2 (2)	C4—C3—H3	120.3
C16—N2—C17	119.9 (3)	C5—C4—Br1	120.0 (3)
C19—N3—Cu1	161.4 (3)	C5—C4—C3	121.0 (3)
H7A—O7—H7B	104.5	C3—C4—Br1	119.0 (3)
C21—O5—H5A	109.5	C15—C14—H14	120.2
N3—C19—S1	178.3 (3)	C13—C14—C15	119.6 (4)
O1—C1—C6	124.2 (3)	C13—C14—H14	120.2
O1—C1—C2	118.7 (3)	C25—C24—Br3	120.1 (3)
C2—C1—C6	117.1 (3)	C25—C24—C23	120.7 (4)
C1—C6—C7	123.2 (3)	C23—C24—Br3	119.2 (3)
C5—C6—C1	119.6 (3)	C14—C13—Br2	119.8 (3)
C5—C6—C7	117.2 (3)	C14—C13—C12	121.5 (3)
N1—C7—C6	125.5 (3)	C12—C13—Br2	118.7 (3)
N1—C7—H7	117.2	C11—C12—H12	120.2
C6—C7—H7	117.2	C13—C12—C11	119.6 (4)
O3—C10—C15	124.0 (3)	C13—C12—H12	120.2
O3—C10—C11	117.7 (3)	C24—C25—H25	119.9
C15—C10—C11	118.2 (3)	C24—C25—C26	120.3 (4)
O4—C18—H18A	110.5	C26—C25—H25	119.9
O4—C18—H18B	110.5	C25—C26—C27	119.6 (4)
O4—C18—C17	106.2 (3)	C21—C26—C25	119.4 (4)
H18A—C18—H18B	108.7	C21—C26—C27	120.9 (4)
C17—C18—H18A	110.5	O5—C21—C26	122.1 (4)
C17—C18—H18B	110.5	O5—C21—C22	118.5 (4)
N2—C17—C18	108.0 (3)	C22—C21—C26	119.3 (4)
N2—C17—H17A	110.1	C24—C23—H23	120.0
N2—C17—H17B	110.1	C22—C23—C24	120.1 (4)
C18—C17—H17A	110.1	C22—C23—H23	120.0
C18—C17—H17B	110.1	C21—C22—H22	119.9
H17A—C17—H17B	108.4	C23—C22—C21	120.1 (4)
C10—C15—C16	123.3 (3)	C23—C22—H22	119.9
C14—C15—C10	120.0 (3)	O6—C27—C26	125.4 (5)
C14—C15—C16	116.7 (3)	O6—C27—H27	117.3
N2—C16—C15	125.4 (3)	C26—C27—H27	117.3
N2—C16—H16	117.3		
			
Cu2—O3—C10—C15	15.2 (4)	C10—C15—C14—C13	1.7 (6)
Cu2—O3—C10—C11	−165.6 (2)	C10—C11—C12—C13	0.8 (6)
Cu2—O4—C18—C17	39.3 (3)	C17—N2—C16—C15	−175.2 (3)
Cu2—N2—C17—C18	31.8 (3)	C15—C10—C11—C12	−0.2 (5)
Cu2—N2—C16—C15	0.8 (5)	C15—C14—C13—Br2	−179.9 (3)
Cu1—O1—C1—C6	−5.0 (5)	C15—C14—C13—C12	−1.0 (6)
Cu1—O1—C1—C2	174.7 (2)	C16—N2—C17—C18	−151.7 (3)
Cu1—O2—C9—C8	−35.9 (3)	C16—C15—C14—C13	−178.5 (3)
Cu1—N1—C7—C6	−0.5 (5)	C2—C1—C6—C7	−178.3 (3)
Cu1—N1—C8—C9	−37.5 (4)	C2—C1—C6—C5	1.1 (5)
Br3—C24—C25—C26	−179.8 (3)	C2—C3—C4—Br1	179.5 (3)
Br3—C24—C23—C22	−179.7 (3)	C2—C3—C4—C5	0.5 (6)
Br2—C13—C12—C11	178.7 (3)	C5—C6—C7—N1	−177.9 (3)
O3—C10—C15—C16	−1.6 (5)	C11—C10—C15—C16	179.1 (3)
O3—C10—C15—C14	178.2 (3)	C11—C10—C15—C14	−1.1 (5)
O3—C10—C11—C12	−179.5 (3)	C8—N1—C7—C6	175.9 (3)
O4—C18—C17—N2	−45.1 (4)	C14—C15—C16—N2	173.2 (3)
O1—C1—C6—C7	1.5 (5)	C14—C13—C12—C11	−0.2 (6)
O1—C1—C6—C5	−179.2 (3)	C24—C25—C26—C21	−0.1 (6)
O1—C1—C2—C3	−180.0 (3)	C24—C25—C26—C27	−179.9 (4)
O2—C9—C8—N1	46.9 (4)	C24—C23—C22—C21	−0.9 (6)
O5—C21—C22—C23	−178.5 (4)	C25—C24—C23—C22	0.4 (6)
C1—C6—C7—N1	1.5 (5)	C25—C26—C21—O5	179.1 (4)
C1—C6—C5—C4	−1.2 (5)	C25—C26—C21—C22	−0.4 (6)
C1—C2—C3—C4	−0.6 (5)	C25—C26—C27—O6	179.7 (5)
C6—C1—C2—C3	−0.2 (5)	C26—C21—C22—C23	0.9 (6)
C6—C5—C4—Br1	−178.6 (3)	C21—C26—C27—O6	−0.1 (8)
C6—C5—C4—C3	0.4 (6)	C23—C24—C25—C26	0.2 (6)
C7—N1—C8—C9	145.7 (3)	C27—C26—C21—O5	−1.1 (7)
C7—C6—C5—C4	178.2 (3)	C27—C26—C21—C22	179.4 (4)
C10—C15—C16—N2	−7.0 (6)		

### (4-Bromo-2-{[(2-hydroxyethyl)imino]methyl}phenololato-κ3O,N,O')(thiocyanato-κN)copper(II)–5-bromo-2-hydroxybenzaldehyde–water (2/1/1) Hydrogen-bond geometry (Å, º)

**Table:**

*D*—H···*A*	*D*—H	H···*A*	*D*···*A*	*D*—H···*A*
O4—H4···O7	0.82	1.75	2.562 (4)	174
O2—H2···O3	0.82	1.84	2.647 (3)	169
O7—H7*A*···O1	0.85	1.88	2.709 (4)	163
O7—H7*B*···S2^i^	0.85	2.64	3.290 (3)	135
O5—H5*A*···O6	0.82	1.94	2.655 (6)	146
C18—H18*A*···Br3^ii^	0.97	3.32	3.856 (4)	117
C18—H18*A*···S1	0.97	2.98	3.801 (4)	143
C18—H18*A*···N3	0.97	3.22	3.785 (5)	119
C17—H17*A*···O5	0.97	2.73	3.368 (5)	124
C16—H16···O6^iii^	0.93	2.52	3.376 (5)	153
C2—H2*A*···O2^iv^	0.93	2.92	3.726 (4)	146
C2—H2*A*···O7	0.93	3.08	3.548 (5)	113
C9—H9*A*···N4^v^	0.97	3.20	3.851 (4)	126
C9—H9*B*···O3^v^	0.97	3.03	3.613 (5)	120
C9—H9*B*···N4	0.97	3.20	3.700 (5)	114
C11—H11···S2^v^	0.93	3.11	3.709 (4)	124
C8—H8*A*···S1^vi^	0.97	3.29	3.881 (3)	121
C8—H8*A*···O3^v^	0.97	3.07	3.773 (5)	130
C8—H8*B*···S2^iii^	0.97	3.30	3.784 (4)	113
C8—H8*B*···O7^vi^	0.97	2.96	3.739 (6)	138
C14—H14···O6^iii^	0.93	3.12	3.827 (6)	134
C12—H12···S2^v^	0.93	2.93	3.616 (5)	131
C22—H22···O6^iii^	0.93	2.97	3.847 (6)	158
C27—H27···Br2^vii^	0.93	3.04	3.949 (6)	166

Symmetry codes: (i) −*x*+1, −*y*+2, −*z*+1; (ii) −*x*, *y*+1/2, −*z*+1/2; (iii) *x*, −*y*+3/2, *z*−1/2; (iv) −*x*+1, *y*+1/2, −*z*+1/2; (v) −*x*+1, −*y*+1, −*z*+1; (vi) −*x*+1, *y*−1/2, −*z*+1/2; (vii) −*x*, −*y*+1, −*z*+1.

## References

[bb1] Abbasi, Z., Salehi, M., Kubicki, M. & Khaleghian, A. (2017). *J. Coord. Chem.* **70**, 3132–3146.

[bb2] Abu-Dief, A. M. & Mohamed, I. M. A. (2015). *Beni-Suef Univ. J. Basic Appl. Sci.* **4**, 119–133. 10.1016/j.bjbas.2015.05.004 PMC7104041 32289037

[bb3] Benito, M., Mahmoudi, G., Molins, E., Zangrando, E., Servati Gargari, M., Gomila, R. M., Frontera, A. & Safin, D. A. (2025). *CrystEngComm* **27**, 164–175.

[bb4] Bruker (2014). *APEX2* and *SAINT*. Bruker AXS Inc., Madison, Wisconsin, USA.

[bb6] Chumakov, M. Yu., Tsapkov, I. V., Bocelli, G., Antosyak, Ya. B. & Gulya, P. (2007). *Crystallogr. Rep.* **52**, 277.

[bb9] De, L. R., Samanta, K., Maiti, K. & Keller, E. (2001). *Inorg. Chim. Acta* **316**, 113–116.

[bb11] Dolomanov, O. V., Bourhis, L. J., Gildea, R. J., Howard, J. A. K. & Puschmann, H. (2009). *J. Appl. Cryst.* **42**, 339–341.

[bb14] Juyal, V. K., Pathak, A., Panwar, M., Thakuri, S. C., Prakash, O., Agrwal, A. & Nand, V. (2023). *J. Organomet. Chem.* **999**, 122825.

[bb16] Krause, L., Herbst-Irmer, R., Sheldrick, G. M. & Stalke, D. (2015). *J. Appl. Cryst.* **48**, 3–10. 10.1107/S1600576714022985 PMC4453166 26089746

[bb17] Liang, F.-Z., Ma, J.-P. & Zhu, J.-H. (2006). *Chin. J. Inorg. Chem.* **22**, 115.

[bb19] More, M. S., Joshi, P. G., Mishra, Y. K. & Khanna, P. K. (2019). *Mater. Today Chem.* **14**, 100195. 10.1016/j.mtchem.2019.100195 PMC7110249 32289101

[bb22] Oshio, H., Hoshino, N., Ito, T. & Nakano, M. (2004). *J. Am. Chem. Soc.* **126**, 8805–8812. 10.1021/ja0487933 15250734

[bb23] Shelar, D. S., Dhavan, P. P., Singh, P. R., Jadhav, B. L., Vaidya, S. P. & Manjare, S. T. (2021). *J. Mol. Struct.* **1244**, 130914.

[bb24] Sheldrick, G. M. (2015). *Acta Cryst.* C**71**, 3–8.

[bb25] Taylor, C. R. & Day, G. M. (2018). *Cryst. Growth Des.* **18**, 892–904. 10.1021/acs.cgd.7b01375 PMC5806084 29445316

[bb26] Thakur, S., Rohilla, J., Sharma, S., Singh, R., Kamboj, R. & Kaur, V. (2024). *Dalton Trans.* **53**, 18283–18295. 10.1039/d4dt02490g 39446127

[bb27] Thakurta, S., Chakraborty, J., Rosair, G., Butcher, R. J. & Mitra, S. (2009*a*). *Inorg. Chim. Acta* **362**, 2828–2836.

[bb28] Thakurta, S., Roy, P., Rosair, G., Gómez-García, C. J., Garribba, E. & Mitra, S. (2009*b*). *Polyhedron* **28**, 695–702.

[bb29] Yu, J. (2011). *Acta Cryst.* E**67**, m1035. 10.1107/S1600536811025852 PMC3212126 22090828

[bb30] Zabierowski, P., Szklarzewicz, J., Kurpiewska, K., Lewiński, K. & Nitek, W. (2013). *Polyhedron* **49**, 74–83.

